# Nanofibrous Polymer Filters for Removal of Metal Oxide Nanoparticles from Industrial Processes

**DOI:** 10.3390/membranes15100291

**Published:** 2025-09-25

**Authors:** Andrzej Krupa, Arkadiusz Tomasz Sobczyk, Anatol Jaworek

**Affiliations:** Institute of Fluid Flow Machinery, Polish Academy of Sciences, Fiszera 14, 80-231 Gdansk, Poland; krupa@imp.gda.pl (A.K.); sobczyk@imp.gda.pl (A.T.S.)

**Keywords:** nanofibrous filter, submicron particles, dust filtration, nanoparticle filtration, PVDF fibrous filter, nanotechnology

## Abstract

Filtration of submicron particles and nanoparticles is an important problem in nano-industry and in air conditioning and ventilation systems. The presence of submicron particles comprising fungal spores, bacteria, viruses, microplastic, and tobacco-smoke tar in ambient air is a severe problem in air conditioning systems. Many nanotechnology material processes used for catalyst, solar cells, gas sensors, energy storage devices, anti-corrosion and hydrophobic surface coating, optical glasses, ceramics, nanocomposite membranes, textiles, and cosmetics production also generate various types of nanoparticles, which can retain in a conveying gas released into the atmosphere. Particles in this size range are particularly difficult to remove from the air by conventional methods, e.g., electrostatic precipitators, conventional filters, or cyclones. For these reasons, nanofibrous filters produced by electrospinning were developed to remove fine particles from the post-processing gases. The physical basis of electrospinning used for nanofilters production is an employment of electrical forces to create a tangential stress on the surface of a viscous liquid jet, usually a polymer solution, flowing out from a capillary nozzle. The paper presents results for investigation of the filtration process of metal oxide nanoparticles: TiO_2_, MgO, and Al_2_O_3_ by electrospun nanofibrous filter. The filter was produced from polyvinylidene fluoride (PVDF). The concentration of polymer dissolved in dimethylacetamide (DMAC) and acetone mixture was 15 wt.%. The flow rate of polymer solution was 1 mL/h. The nanoparticle aerosol was produced by the atomization of a suspension of these nanoparticles in a solvent (methanol) using an aerosol generator. The experimental results presented in this paper show that nanofilters made of PVDF with surface density of 13 g/m^2^ have a high filtration efficiency for nano- and microparticles, larger than 90%. The gas flow rate through the channel was set to 960 and 670 l/min. The novelty of this paper was the investigation of air filtration from various types of nanoparticles produced by different nanotechnology processes by nanofibrous filters and studies of the morphology of nanoparticle deposited onto the nanofibers.

## 1. Introduction

Filtration of nanoparticles and submicron particles is an important problem in air conditioning and ventilation systems due to the presence of submicron particles comprising fungal spores, bacteria, viruses, tobacco smoke, and microplastics produced by the clothes’ wearing. Industry can produce fine particles, smaller than 1 µm, in various nanotechnology processes. Due to their specific physicochemical properties and high surface area-to-volume ratio, nanoparticles containing different compounds of metals or metalloids are widely used in various industries, for modern nanotechnology processes, for manufacturing different products, and for research purposes in material science and engineering, environmental science, medicine, or biotechnology. These particles can be released to the environment during the process of their synthesis, deposition, dispersion, manufacturing, transportation, or disposal, or as an effect of their accidental leakage during manufacturing or transportation. Submicron and nanoparticles dispersed in the aerosol phase, in particular, compounds of metals and metalloids, are dangerous to human and animal health after their penetration into the lower respiratory tract and blood system. Such particles have strong cytotoxicity and genotoxicity in contact with cell membranes [1,2,3,4,5,6,7,8]. The results presented by Minigalieva et al. showed that TiO_2_, Al_2_O_3_ and SiO_2_ nanoparticles caused significant toxic damage of kidneys, liver, and spleen in rats, and enhanced genomic DNA fragmentation [9]. Among these nanoparticles, aluminum oxide proved to be the most noxious. Wei et al. proved that the attachment of metal oxide nanoparticles to cell membranes may lead to cytotoxicity due to various physical interactions [10]. The most dangerous were Al_2_O_3_ and SiO_2_ nanoparticles, which destroyed an oppositely charged cellular membrane by electrostatic attraction and forming a larger amount of hydrogen bonds with the carbonyl and phosphate groups of phospholipids of cell membranes.

After an industrial process, nanoparticles can be retained in a conveying gas, and the gas must be cleaned before it is released into the atmosphere. Filtration is a commonly used technology for the post-processing gas cleaning from nanoparticles, before this gas can be discharged to the atmosphere. However, submicron and nanoparticles usually slip throughout voids between the fibers of conventional filters, and the fractional filtration efficiency for these particles is significantly lower than for larger particles [11,12,13,14]. It was estimated that about 1.5% of nanoparticles used in various technologies is released to the atmosphere [15]. Although these nanoparticles comprise less than 5% of the total emission of all kinds of nanomaterials to the environment [15] (to air, soil, and water), their negative health effects during inhalation are more dangerous than those released into the soil or groundwater. For these reasons, further research is required to obtain sufficiently high filtration efficiency of fibrous filters used for nano- and submicron particles removal.

A solution to this problem can be nanofibrous filters. Various methods of making nanofibrous filtration mats, such as centrifugal spinning, melt spinning, wet spinning, melt blowing, and spun bonding have been developed, which are applied in different nanotechnologies, for example, tissue scaffolds, drug delivery systems, biosensors, and filters [16,17]. The most effective method for the manufacturing of nanofibrous filters is electrospinning (electrohydrodynamic spinning) of a polymer material [18,19,20,21,22,23,24,25,26,27,28,29]. The physical basis of the electrospinning process is the employment of electrical forces to create a tangential stress acting on the surface of viscous liquid flowing out from a capillary nozzle. The jet is pulled out under this stress, and eventually a thin fiber with a diameter of 100–500 nm is formed after solvent evaporation. During electrospinning, the polymer solution is pumped under a low pressure to the nozzle with a stable flow rate of the order of 1 mL/h. The velocity of the polymer nanofiber during its extraction increases from 2 m/s to 200 m/s in the interelectrode space, depending on the physical properties of the solution and the fabrication conditions [26]. The produced fibers are collected on a flat metal substrate or a metal mesh to form a thin nanofibrous filtration mat. Filters produced by electrospinning are made of fibers of similar diameters, and are characterized by a high homogeneity of their structure [29].

Abdulhamid and Muzamil [30] analyzed the publication dynamics in the field of electrospinning in the years from 2000 to 2021 using the Scopus database, and the keyword: ‘electrospun nanofiber’. The authors noticed an almost exponential increase in the number of publications, which attained a number exceeding 1800 in 2021. Most of the papers published in the field of electrospinning concern the fabrication of nanofibers in various nanotechnologies, with an increasing number of biotechnology applications [31,32]. Recently, there have been many papers published on the fabrication of nanocomposite membranes for gas filtration [33,34,35] or liquid separation [28].

Polymer nanofibrous filters are used for the removal of nanoparticles from gases and water, mainly for waste water treatment, water-in-oil separation, or desalination [36,37,38,39,40,41,42]. The experiments with the filtration of SiO_2_, TiO_2_, and ZnO nanoparticles in water, using Al_2_O_3_ ceramic filters, were carried out by Le et al. [43].

The research into the field of nanofibrous filters application in the air cleaning was carried out in [44,45,46,47,48]. The authors investigated the efficiency of filtration of particulate pollutants by nanofibrous filters produced by a polymer blowing method. Podgórski and Bałazy [46] proposed a model for the motion and deposition of particles on a filter. Podgórski investigated the process of particle penetration through a heterogeneous nanofibrous filter for the filtration of aerosol composed of nanoparticles [47]. The results of measurements of dust filtration efficiency by nanofibrous filters produced by polymer blowing were presented by Przekop and Gradoń [44]. However, the blowing technology does not offer the possibility to produce nanofibers with a controlled diameter and controlled degree of alignment. Sambaer et al. [49,50,51] presented a 3D model of filtration by a polyurethane nonwoven nanofibrous filter produced by electrospinning. The fractional filtration efficiency was minimal in the particle size range between 60 and 200 nm. This filtration efficiency changed from about 40 to 90%, depending on the gas velocity, which was decreased from 0.085 m/s to 0.02 m/s, respectively. The authors revealed that the particle–fiber friction coefficient has a significant role in the filtration efficiency, especially for nanoparticles smaller than 200 nm. The slip effect in the nanofibrous filters was simulated by Pan et al. [52]. The simulations showed that for nanofibers with diameters close to the mean free path of gaseous molecules, the nanoparticles tend to slip through the filter, particularly at low pressures (i.e., relatively low pressure, and the pressure drop is smaller than 15%). The slip effect improves filtration efficiency for particles larger than 100 nm.

Research on the use of nanofibrous filters in the field of gas filtration is not well represented in the literature. The Web of Science Core Collection returns 41 titles on the query “TS = (polymer nanofibers) AND TS = (nanoparticle filtration)”. A comprehensive review of electrospun nanofiber membranes application to air filtration was provided by Zhou et al. [16]. Measurements of the filtration efficiency for nanoparticles by nonwoven filters have been reported by Heim et al. [53], Bałazy et al. [54], Japuntich et al. [55], Kim et al. [56], Podgórski et al. [57], Steffens and Coury [58], Wang et al. [59], Su et al. [60,61], and Lima et al. [29].

This paper presents results of a study of filtration efficiency of submicron and nanoparticles by nonwoven nanofibrous filter, with a potential application of this process in nanotechnology or other post-processing gas cleaning systems. The goal of these studies was to investigate the nanoparticle morphology deposited onto the nanofibers, instead of investigation of properties of dust cake in the bulk. This should allow better understanding of the filtration mechanisms by nanofibrous filters. The novelty of these experiments was the application of nanofibrous filters for air filtration of nanoparticles used in technology processes. Similar experiments with other particles were carried out, for example, by Lima et al. [62], which have filtered Fe_3_O_4_, ZnO, TiO_2_, and CeO_2_ nanoparticles from the gas flow by a nanofibrous electrospun filter. Tok and Ertekin [63] used various composite nanofibrous filters for the filtration of Fe_2_O_3_ and ZnO nanoparticles. The current experimental studies cover the filtration of TiO_2_, MgO, and Al_2_O_3_ nanoparticles with a mean particle size below 1 µm. These particles were selected because the oxides of these metals are the most abundant in some production processes. For example, in the post-processing gas from aluminum–titanium alloy production, Minigalieva et al. [9] demonstrated that the percentage of each of these metals within all metals constituting the aerosol particles in this process (excluding oxygen and carbon content in the post processing gas) was higher than 10%. In this research, the nanoparticle aerosol was produced by an aerosol generator, which atomized a suspension of commercially available nanoparticles in methanol. The filtration efficiency measurements were carried out in a small laboratory duct with an inner diameter of 18 mm.

## 2. Materials and Methods

**Figure 1:**
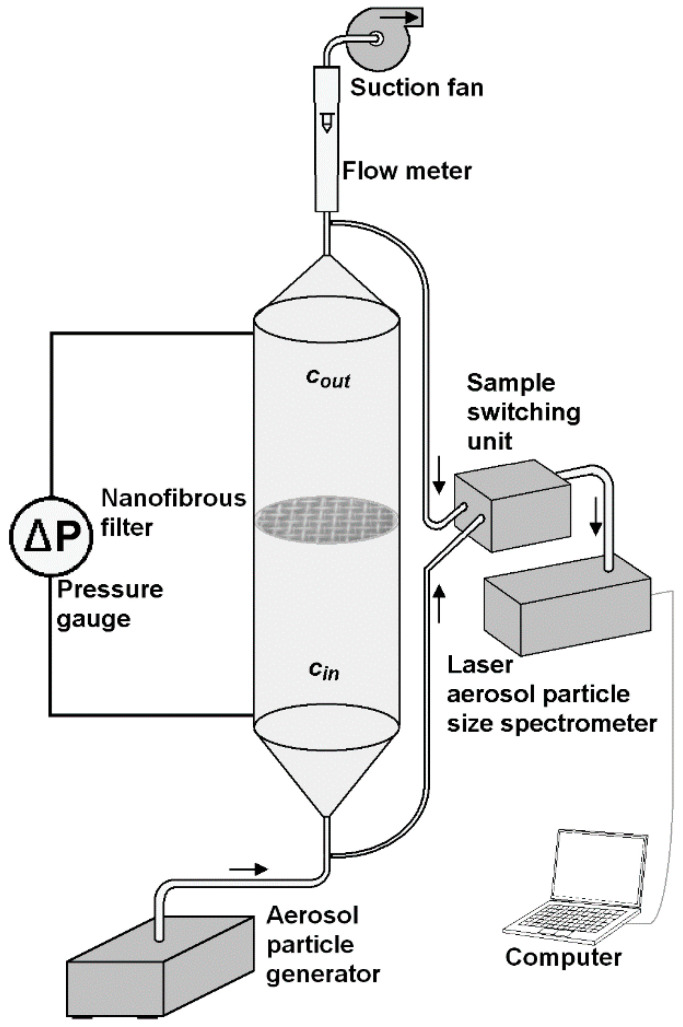
Laboratory channel for the measurement of particle filtration efficiency by a polymer nanofibrous filter.

A schematic of the laboratory set-up, with a channel designed for measuring the gas filtration efficiency of submicron particles, is shown in Figure 1.

The following operations were conducted in each series of measurement:

1. Cleaning the channel by its washing with distilled water and drying;
2. Mounting a frame with virgin filter within the channel;
3. Closing the channel with the connectors equipped with elastic hose;
4. Switching-on the outlet fan with desired flow rate;
5. Measurement of the pressure drop across the clean filter;
6. Switching-on the particle generator;
7. Recording the particle size distribution at the inlet/outlet by a computer for a 5 min;
8. Switching off the particle generator;
9. Measurement of the pressure drop across the loaded filter after 5 min;
10. Switching-off the outlet fan;
11. Dismounting the connectors with elastic hose;
12. Dismounting the frame with loaded filter;
13. Inspection the filter under SEM.

The experiments were carried out in a vertical channel of circular cross-section of an inner diameter of 18 mm, and a length of 200 mm, made of PMMA. The aerosol was produced by atomization of a suspension of TiO_2_, MgO, and Al_2_O_3_ nanoparticles using ATM 226 (TOPAS GmbH, Dresden, Germany) aerosol generator. The mass concentration of these particles in methanol was 0.05%. Dynasylan^®^ Memo (Hanau, Germany) was added to this suspension in order to stabilize the solution. The ATM 226 aerosol generator complies with the VDI-directive 3491. The aerosol was injected at the bottom of the channel with a flow rate of 300 l/h, and flowed upwards. In the half-height of the channel a circular frame with a nanofibrous filter was mounted. The nanoparticles of MgO (of mean size 100 nm, material density of 3.58 g/cm^3^), anatase TiO_2_ (32 nm, 3.9 g/cm^3^), and γ-Al_2_O_3_ (60 nm, 3.965 g/cm^3^), were delivered by Alfa Aesar (Karlsruhe, Germany).

The nanofibrous filters were produced by the method of electrospinning using a capillary nozzle of inner diameter of 0.4 mm and outer diameter of 0.5 mm. The voltage of +13.5 kV was applied to the nozzle from high-voltage power supply SL300 30 kV (SPELLMAN, Hauppauge, NY, USA). The distance between the nozzle outlet and the scaffold mesh, on which the fibers were deposited, was 120 mm. The concentration of polyvinylidene fluoride (PVDF, Mw = 455,000 g/mol, Sigma-Aldrich, St. Louis, MO, USA) polymer dissolved in a mixture of 47 wt.% dimethylacetamide (DMAC, Sigma-Aldrich, St. Louis, MO, USA) and 53 wt.% acetone (Chempur, Piekary Śląskie, Poland) was 15 wt.%. The flow rate of polymer solution fed by the Fusion 200 High Precision Dual syringe pump (CHEMYX Inc., Stafford, TX, USA) was 1 mL/h. The solution was stirred by a magnetic stirrer for a time of 12 h at room temperature. The spinning was carried out at room temperature (20–21 °C) and humidity of 45–50%. The method for fabricating nanofibrous filters was described in previous articles [64,65]. The nanofibers were deposited onto a grounded mesh made of a stainless steel wire of 0.25 mm in diameter. A new clean nanofibrous filter was used for each measurement.

PVDF was used in this research due to its specific properties, such as excellent thermal and chemical stability, ferroelectric dipole interaction, excellent mechanical strength, low reactivity, excellent processability, and high hydrophobicity [66]. High air filtration efficiency by low pressure drop can be obtained for such filters. For example, Bui et al. [67] obtained a 97.4% filtration efficiency of PVDF filter for PM0.3 particles by 51 Pa pressure drop. PVDF has also a high dielectric constant (larger than 50) that allows obtaining greater adhesion force during the filtration of dielectric particles, or by its operation as electrostatic hybrid filter. Such a hybrid filter can operate at lower voltages by the same filtration efficiency [68].

The aerosol flowed through the channel under the action of a suction pump connected at the upper part of the channel. Two sampling connectors were mounted upstream and downstream of the filter to measure the particle size distribution and their concentration at the inlet and outlet of the channel by laser aerosol particle size spectrometer LAP 322 (TOPAS GmbH, Dresden, Germany). The device measures the size of particles in a range from 225 nm to 10 μm, in 64 size classes; therefore, the collection efficiency for particles of a size smaller than 225 nm were not measured. The aerosol samples were transported from both connectors to the spectrometer via sample switching unit SYS520 (TOPAS GmbH, Dresden, Germany). The air flow rate was measured using a rotameter RTV-10-300 (Rotametr, Gliwice, Poland). Two connectors were also mounted in the channel wall for measuring the pressure drop across the nanofibrous filter. The pressure drop across the nanofibrous filter was measured by pressure gauge TESTO 512 (TESTO, Titisee-Neustadt, Germany), in a range from 0 to 200 Pa (resolution 0.1 Pa), and 0 to 20,000 Pa (10 Pa).

Raman spectra were used to determine qualitatively the ratio of β and α phases in PVDF nanofibers and to characterize the polar properties of the obtained filter. The Raman spectra of the electrospun PVDF nanofibers were determined using confocal micro-Raman spectrometer (InVia, Renishaw, Wotton-under-Edge, UK) with a 100× objective lens and a laser emission at a wavelength of 514 nm, and operating at 10% of its total power (50 mW).

The fractional filtration efficiency of the PVDF nanofibrous filters for the investigated nanoparticles was determined from the size distribution at the inlet and outlet of the channel measured by particle size spectrometer LAP322, using the following equation:

(1) $$\eta \left(i\right)=\left(1-\frac{c_{out(i)}}{c_{in(i)}}\right)100\%,$$

where *c*_*out*(*i*)_ and *c*_*in*(*i*)_ are the particle number concentrations of the *i*-th fraction of particles at the outlet and the inlet of the filter, respectively.

Additionally, the filtration efficiency at 400 nm (according to EN 779:2021) was calculated for these nanofibrous filters. Morphological studies of the particles and the contaminated nanofibrous filters were carried out using Scanning Electron Microscope (SEM) EVO 40 (ZEISS, Oberkochen, Germany).

The gas flow rate through the channel was set to 960 or 670 l/min that corresponded to a velocity of 1 m/s and 0.7 m/s, respectively. The filter hydrodynamic characteristics, i.e., the pressure drop across the filter vs. air flow velocity were measured for the case of clean filter, prior to the aerosol particle dispersion, and the same characteristics were measured after 5 min of deposition of the particles on the filter. This short time was selected to observe the behavior of individual particles after their filtration and analyze their morphology. An increase in the filter resistance was determined from the difference of both pressure drops. The contaminated filter was removed from the channel and examined under SEM. The same measurement procedure was applied to each type of particle.

## 3. Results

### 3.1. Nanofibrous Filter Properties

**Figure 2:**
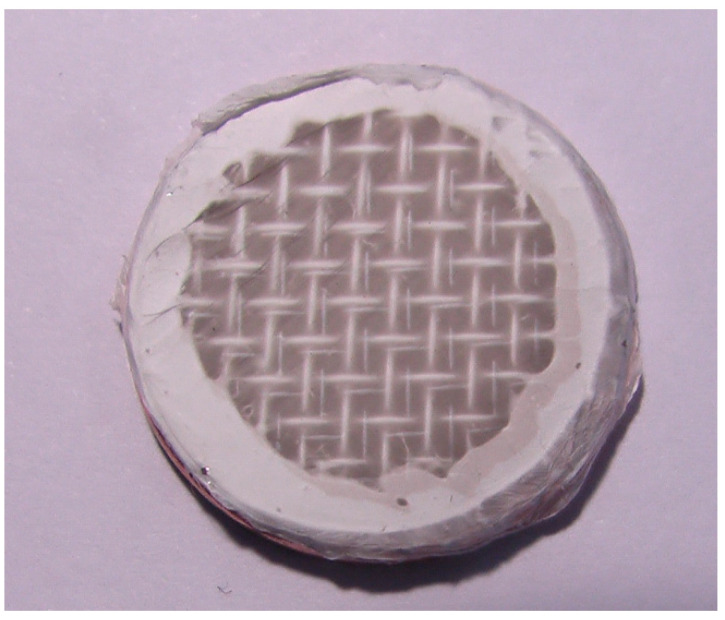
Frame with metal mesh and electrospun PVDF nanofibers. Deposition time 10 min. Flow rate of polymer solution 1 mL/h. PVDF concentration 15 wt.% in DMAC and acetone mixture. The nanofiber layer is almost transparent and the metal mesh underneath can be noticed.

Figure 2 shows a photograph of a nanofibrous filter made of PVDF nanofibers deposited onto a metal mesh, supported by a circular PMMA frame. Nanofibrous filters, used in the following way, were produced by electrospinning of PVDF polymer onto a stainless steel mesh for a time of 10 min.

**Figure 3:**
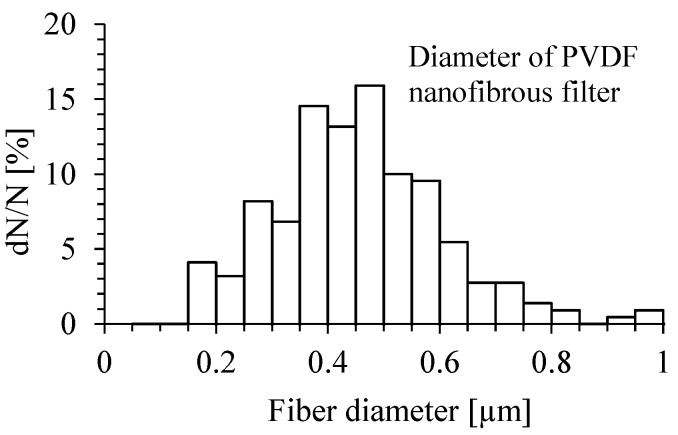
Diameter distribution of PVDF nanofibers. The mean value of nanofiber diameter = 411 nm, the median diameter = 404 nm, and standard deviation = 175 nm.

The distribution of the diameter of PVDF nanofibers of the filter is presented on Figure 3. The diameter of PVDF nanofibers determined from SEM micrographs ranged from 150 nm to 850 nm.

The weight of PVDF nanofibers accumulated on the mesh, estimated from the time of electrospinning and the solution flow rate, was about 2.2 mg, and the surface density was 13 g/m^2^. The mean value of porosity of nanofibrous filter was determined from the SEM micrographs to be about 40%, using the method described in the paper [65] (cf. also [69]).

The filter thickness *δ* can be determined from its specific weight and packing density:

(2) $$\delta =\frac{W}{\rho \cdot \alpha }$$

where *W* is the specific weight of the filter (kg/m^2^), *ρ* is the density (kg/m^2^) of the fiber’s material, and *α* is the filter packing density (%) [70,71]. The packing density is defined as the percentage of the space occupied by the fibers, and can be determined from the equation:

(3) α=100−ε

where *ε* is the filter porosity (%).

For PVDF density 1780 kg/m^3^, specific weight 0.013 kg/m^2^, and estimated porosity of 40%, the filter thickness can be determined to be about 12 µm. For the mean fiber diameter of 400 nm, it can be estimated that the nanofibrous filter is formed by 30 layers.

There are five crystalline phases of PVDF: α-phase, β-phase, γ-phase, δ-phase, and ε-phase [72]. The non-polar α-phase is the most thermodynamically stable. It characterizes by alternating trans and gauge linkage (TGTG’) conformation. The β-phase is the phase with the highest dipolar moment and has an all-trans (TTT) planar zigzag conformation [72]. Electrospinning is a technique that produces PVDF nanofibers with a high β-phase fraction and crystallinity by aligning molecular dipoles (–CH_2_ and –CF_2_) along an applied electric field direction [73] and mechanical force [74].

**Figure 4:**
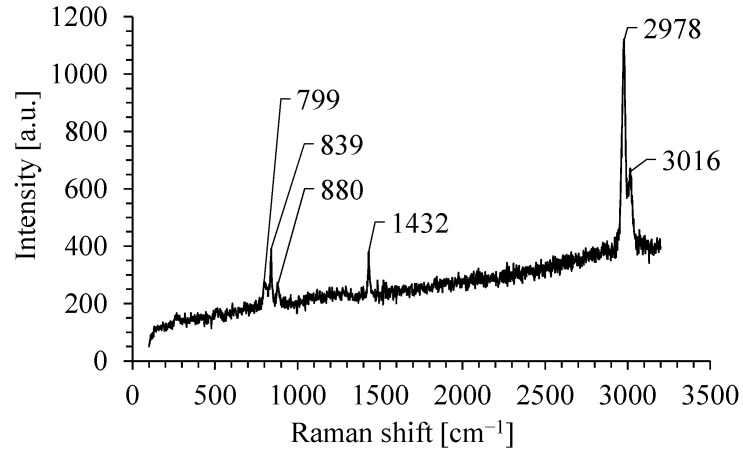
The Raman spectra of PVDF nanofibrous filter.

Typical Raman spectrum of PVDF nanofibrous filter in the range from 150 cm^−1^ to 3200 cm^−1^ is shown in Figure 4. The Raman spectrum of nanofibrous filter consists of a few bands.

The band at about 804 cm^−1^ is assigned to the rocking motion of CH_2_ and it is a typical band for α-phase-rich PVDF. The band at about 839 cm^−1^ is caused by CH_2_ rocking and CF_2_ symmetric stretching and it is typical for β-phase. The band at about 880 cm^−1^ is assigned to the CC symmetric and antisymmetric stretching and CF_2_ symmetric stretching and is attributed to both phases. The band at 1432 cm^−1^ is caused by bending CH_2_ vibrations [75]. The band at 2978 cm^−1^ is usually attributed to CH_2_ symmetric stretching, and the band at 3016 cm^−1^ to CH_2_ antisymmetric stretching [76]. These two bands are commonly associated with the β-phase.

The ratio between the intensities of β and α phases in PVDF nanofibers can be calculated from the following formula [75]:

(4) $$\frac{\beta_{phase}}{\alpha_{phase}}=\frac{A_{839}}{A_{799}}$$

where A_839_ and A_799_ are the areas of the bands at 839 and 799 cm^−1^, respectively. The area was calculated using the Lorentzian profile for these bands.

The ratio between the β and α phases in PVDF nanofibrous was about 1.2, and it could be supposed that the obtained nanofibers of this filter are partially polar, which enhances the filtration efficiency of the nanofibrous filter.

### 3.2. TiO_2_ Nanoparticle Filtration

Titanium dioxide has twelve polymorph forms, and three of them, Rutile (tetragonal crystal system), Anatase (tetragonal), and Brookite (orthorhombic), are mostly applied in nanotechnology. TiO_2_ is a medium bandgap material, of high dielectric constant (20), high thermal stability, and high refractive index. TiO_2_ is used as an additive to paints, textiles, cosmetics, and paper. TiO_2_-covered surfaces possess anti-fogging, self-cleaning, and UV shielding properties. TiO_2_ is also used in the production of solar cells, catalysts, gas sensors, and rechargeable batteries [77,78,79,80]. Photocatalytic microorganisms inactivation [81,82], photocatalytic degradation of chemicals [83], cements [84], geopolymer composites [85], optical glasses [86], plant fertilizers [87], anti-corrosion and hydrophobic coatings [88], antimicrobial coatings [89], nanocomposite membranes of anti-fouling properties [43,90,91], and optoelectronic devices [92] are other applications of TiO_2_. Physical properties of TiO_2_ nanoparticles and their different applications in nanotechnology have been recently reviewed by Shivani et al. [93]. Perspectives of TiO_2_ nanoparticles application to energy conversion devices were discussed by Baghali et al. [22].

**Figure 5:**
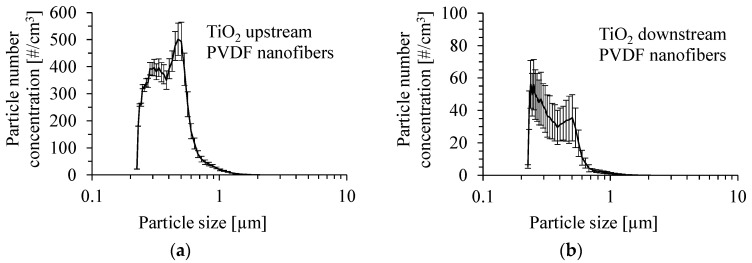
Number size distribution of TiO_2_ particles produced by atomization of a suspension of 0.05 wt.% of TiO_2_ particles in methanol with the addition of Dynasylan^®^ Memo (**a**) and downstream of the PVDF nanofibrous filter (**b**). Gas velocity 1 m/s. Mean inlet mass concentration of particles 1.8 mg/m^3^.

The number size distribution of anatase TiO_2_ particles produced by the atomization of a suspension of 0.05 wt.% of these particles in methanol with the addition of Dynasylan^®^ Memo is shown in Figure 5a. The mean inlet mass concentration of particles was 1.8 ±0.26 mg/m^3^. The size distribution of TiO_2_ particles after passing through the PVDF nanofibrous filter for a gas velocity of 1 m/s is presented in Figure 5b. The size distribution of TiO_2_ particles was determined as the average value from several runs. The number mean diameter of particles upstream of the PVDF nanofibrous filter was 400 nm, and the Sauter mean diameter was 549 nm. Downstream of the PVDF nanofibrous filter, the number mean diameter of particles and the Sauter mean diameter were 356 and 493 nm, respectively.

**Figure 6:**
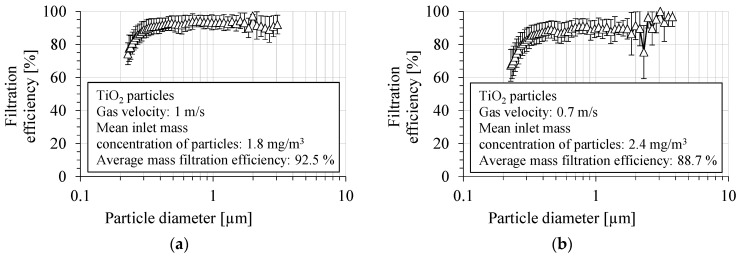
Fractional filtration efficiency of PVDF nanofibrous filter for TiO_2_ particles for gas velocity: (**a**) 1 m/s, (**b**) 0.7 m/s. Fractional filtration efficiency is about 90%, but it decreases for particles smaller than 300 nm due to higher particle penetration through the voids in nanofibrous filter.

The fractional filtration efficiency for TiO_2_ particles by the PVDF nanofibrous filter is shown in Figure 6a,b, for two aerosol velocities: 1 m/s and 0.7 m/s, respectively. The filtration efficiency was determined as the average value from several runs. The fractional filtration efficiency was determined in the particle size range from 225 nm to 3 μm. The filtration efficiency of the PVDF nanofibrous filter was about 93.0 ± 3.8% and 89.2 ± 5.8% in the particle size range above 300 nm, for a gas velocity of 1 and 0.7 m/s, respectively. The overall number filtration efficiency in the entire size range measured was 89.6 ± 4.4% for a gas velocity of 1 m/s, and 84.8 ± 6.7% for a gas velocity of 0.7 m/s. The filtration efficiency was about 91.8 ± 3.5% and 88.0 ± 5.6%, for particles of a diameter of 400 nm, for a gas velocity of 1 m/s and 0.7 m/s, respectively.

**Figure 7:**
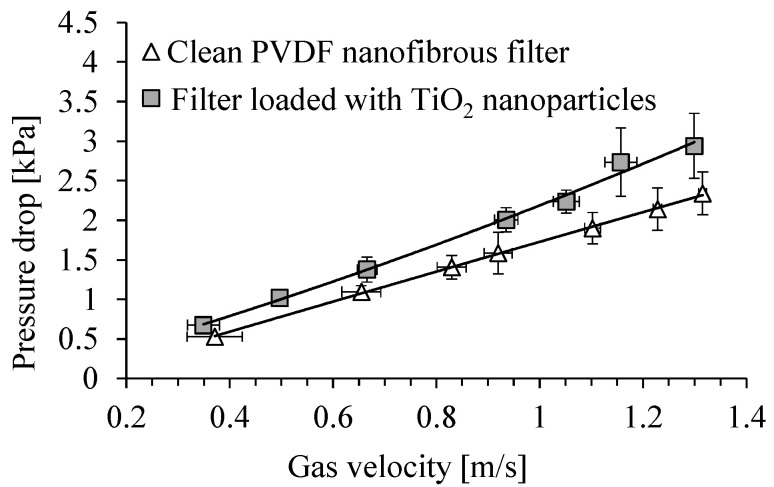
Dependence of pressure drop across PVDF nanofibrous filter on the gas velocity. Nanofibrous filter with TiO_2_ particles after 5 min of filter operation (filter load 520 mg/m^2^).

Figure 7 shows the dependence of the pressure drop across a clean PVDF nanofibrous filter on the gas velocity, and for the same filter loaded with TiO_2_ particles, after a time of 5 min of filter operation. The total filter load was 520 mg/m^2^. The pressure drop across the clean filter was about 1.7 kPa, for a gas velocity of 1 m/s, and it increased to about 2.2 kPa, after filter load with TiO_2_ particles. For a velocity of 0.7 m/s, the pressure drop increased from about 1.2 kPa to 1.5 kPa.

**Figure 8:**
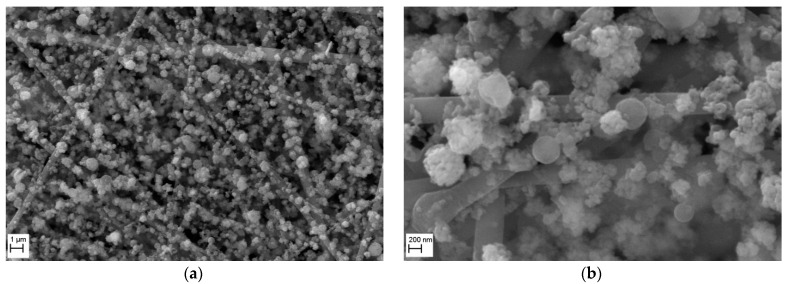
SEM micrographs of the surface of PVDF nanofibrous filter. Nanofibrous filter with TiO_2_ particles after 5 min of filter operation for different magnifications: (**a**) 10,000×, (**b**) 50,000×. Total filter load 520 mg/m^2^. The fibrous filter is uniformly covered with the particles. The TiO_2_ particles are deposited on the fibers in the form of chain-like agglomerates. Some voids are also occupied.

The morphology of a nanofibrous filter covered with TiO_2_ particles after 5 min of operation, investigated under SEM, is shown in Figure 8 for two magnifications. The micrograph obtained at a magnification of 10,000× (Figure 8a) shows that the surface of filter is uniformly covered with particles, indicating a high homogeneity of the filter produced by electrospinning. The micrograph taken at a magnification 50,000× (Figure 8b), presents a close-up view of TiO_2_ particles deposited onto the fibers and in the voids between them. The TiO_2_ particles formed the chain-like aggregates. Some larger particles (about 500 nm) of nearly spherical shape formed aggregates of a collector type, with smaller particles (<100 nm) attached to their surface.

### 3.3. MgO Nanoparticle Filtration

Magnesium oxide is a wide-bandgap material with great thermodynamic stability, a low refractive index, and a relatively low dielectric constant (3.2 to 9.9)—compared to other metal oxides [94,95], which is used as a component of catalysts [80], flame retardant [96], and protective-coating materials [97]. It is also used as a component of glasses [98], ceramics [99], and energy storage devices [100,101]. MgO is also applied as an antimicrobial agent in textile and leather products [102], or for soil amelioration [103]. Various applications of MgO nanoparticles in nanotechnology have been reviewed by Duan et al. [96] and Nguyen et al. [104], and in nanomedicine by Fahmy et al. [105].

**Figure 9:**
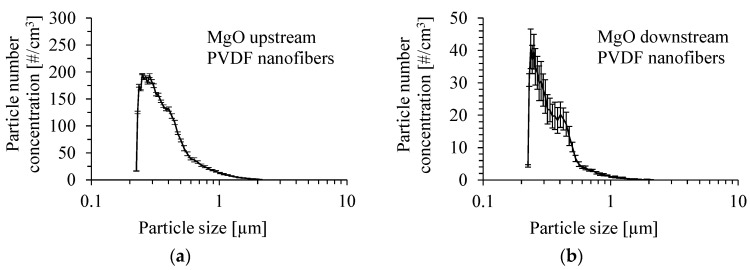
Number size distribution of MgO particles produced by atomization of a suspension of 0.05 wt.% of MgO particles in methanol with the addition of Dynasylan^®^ Memo (**a**) and downstream of the PVDF nanofibrous filter (**b**). Gas velocity of 1 m/s. Mean inlet mass concentration of particles: 0.85 mg/m^3^.

The number size distribution of MgO particles produced by the atomization of a suspension of 0.05 wt.% of these particles in methanol with the addition of Dynasylan^®^ Memo is shown in Figure 9a. The size distribution of MgO particles after passing through the PVDF nanofibrous filter by a gas velocity of 1 m/s is presented in Figure 9b. The size distribution was determined as the average value from several runs. The mean inlet mass concentration of particles was 0.85 ± 0.07 mg/m^3^. The number mean diameter of particles upstream of the PVDF nanofibrous filter was 376 nm, and the Sauter mean diameter was 732 nm. Downstream of the PVDF nanofibrous filter, the mean diameter of particles and Sauter mean diameter were 343 nm and 583 nm, respectively.

**Figure 10:**
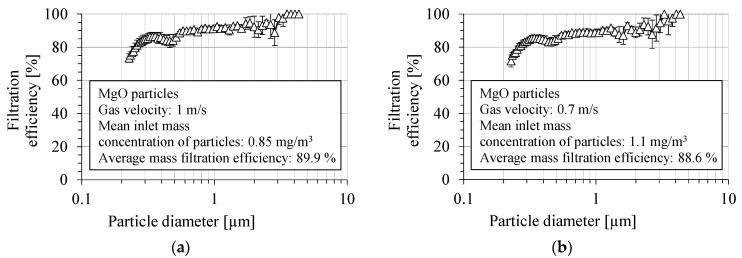
Fractional filtration efficiency of PVDF nanofibrous filter for MgO particles for gas velocity: (**a**) 1 m/s, (**b**) 0.7 m/s. Fractional filtration efficiency is about 80–90% and approaches 100% for particles larger than 3 µm, but becomes smaller for the particles smaller than 300 nm due to higher particle penetration through the voids of nanofibrous filter.

The fractional filtration efficiency for MgO particles by the PVDF nanofibrous filter is presented in Figure 10a,b, for two aerosol velocities: 1 m/s and 0.7 m/s, respectively. The filtration efficiency was determined as the average value from several measurements. The fractional filtration efficiency was determined in the particle size range from 225 nm to 4 μm. The overall filtration efficiency of the PVDF nanofibrous filter was about 90.4 ± 2.1% and 89.0 ± 0.8%, in the particle size range above 300 nm, and for a gas velocity of 1 and 0.7 m/s, respectively. For a gas velocity of 1 m/s, the average number filtration efficiency was about 83.8 ± 2.9%, and for the velocity of 0.7 m/s, it was 82.9 ± 1.5%. The filtration efficiency was about 85.3 ± 2.9% for particles with a diameter of 400 nm, for a gas velocity of 1 m/s, and 84.2 ± 2.4% for 0.7 m/s.

**Figure 11:**
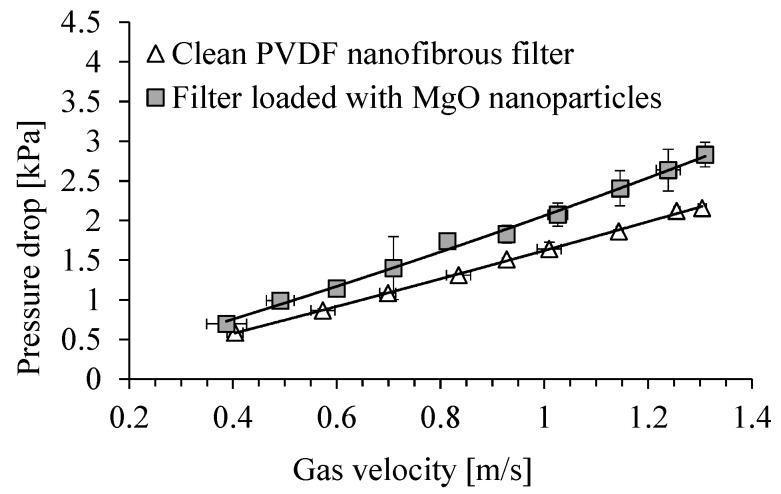
Dependence of the pressure drop on PVDF nanofibrous filter on the gas velocity. Nanofibrous filter with MgO particles after 5 min of filter operation. Total filter load 243 mg/m^2^.

The dependence of the pressure drop across the clean PVDF nanofibrous filter on the gas velocity and for the filter contaminated with MgO particles is shown in Figure 11. The time of filter operation was 5 min. The total filter load was 243 mg/m^2^. Because of filling the voids between the fibers of filter, the filter resistance increased during the filter operation. The pressure drop increased from about 1.4 kPa for the clean filter to 2.1 kPa, after its contamination with MgO particles, for a gas velocity of 1 m/s. For a gas velocity of 0.7 m/s, the pressure drop increased from 1.0 kPa to 1.4 kPa.

**Figure 12:**
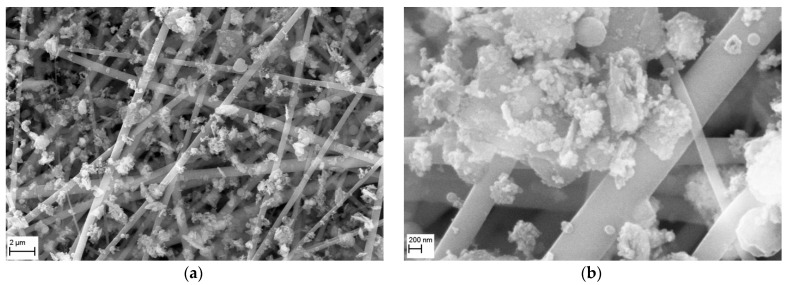
SEM micrographs of the surface of PVDF nanofibrous filter with MgO particles after 5 min of filter operation for different magnifications: (**a**) 10,000×, (**b**) 50,000×. Total filter load 243 mg/m^2^. The particles are mainly deposited on the fibers of fibrous filter. The MgO particles are deposited on the fibers in the form of agglomerates, which can bridge the voids.

The morphology of a nanofibrous filter covered with MgO particles, after 5 min of operation is shown in Figure 12, for two SEM magnifications. The micrograph obtained at a magnification of 10,000× (Figure 12a) shows that the particles are mainly deposited on the fibers of this filter, with only a small number of particles filling the filter’s voids. The micrograph taken at 50,000× magnification (Figure 12b) presents a close-up view of MgO particles deposited onto the fibers, which formed large aggregates, sometimes bridging the fibers. The collector type aggregates, comprised of larger MgO particles covered with particles < 100 nm, can also be observed.

### 3.4. Al_2_O_3_ Nanoparticle Filtration

Aluminum oxide has exceptional material properties, such as high strength-to-density ratio, high corrosion resistance, high flame resistance, high melting point, high chemical stability, medium dielectric constant (9), high electric resistance, wide bandgap, and low thermal conductivity. Al_2_O_3_ nanoparticles are used as an additive to absorbers of various water pollutants [106,107], nanoporous filtration membranes [43,108], antifouling coating of nanocomposite membranes [90,91], nanoporous filters for liquid metals [109], optoelectronic devices [110], optical glasses [111], anti-reflection coatings [112], catalyst supports [113], a component of paints and surface coating layers [114], cement-based building materials [85], and geopolymers [115].

**Figure 13:**
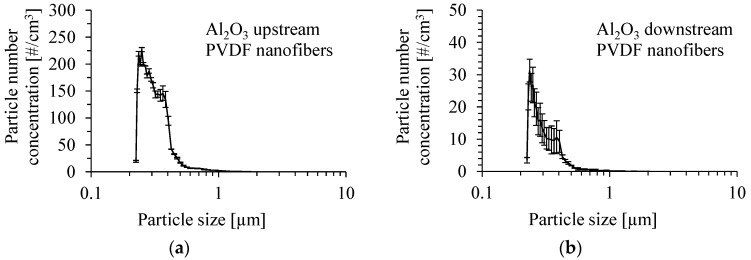
Number size distribution of Al_2_O_3_ particles produced by atomization of a suspension of 0.05 wt.% of Al_2_O_3_ particles in methanol with the addition of Dynasylan^®^ Memo (**a**), and downstream of PVDF nanofibrous filter (**b**). Gas velocity 1 m/s. Mean inlet mass concentration of particles 0.28 mg/m^3^.

The number size distribution of γ-Al_2_O_3_ particles produced by the atomization of their suspension in methanol, with the addition of Dynasylan^®^ Memo, is shown in Figure 13a, for a concentration of 0.05 wt.%. The number mean diameter of the particles was 314 nm, and the Sauter mean diameter was 428 nm at the upstream of the PVDF nanofibrous filter. The mean inlet mass concentration of particles was 0.28 ± 0.1 mg/m^3^. The number size distribution of Al_2_O_3_ particles downstream of the PVDF nanofibrous filter is shown in Figure 13b for a gas velocity of 1 m/s. The number mean diameter of particles at the outlet and the Sauter mean diameter were 302 and 400 nm, respectively.

**Figure 14:**
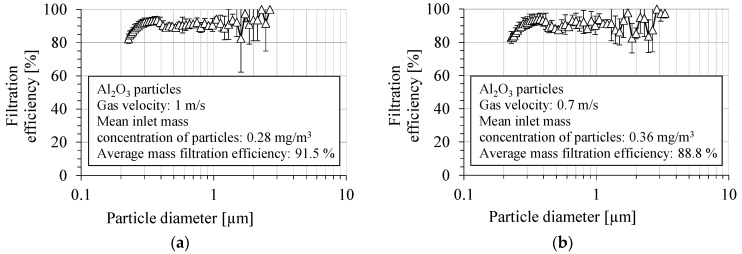
Fractional filtration efficiency of PVDF nanofibrous filter for Al_2_O_3_ particles for gas velocity: (**a**) 1 m/s, (**b**) 0.7 m/s. Fractional filtration efficiency oscillates between 80% and 100%, and it decreases below this range for the particles smaller than 300 nm due to higher particle penetration through the voids in nanofibrous filter.

The fractional filtration efficiency of the PVDF nanofibrous filter for Al_2_O_3_ particles is shown in Figure 14a,b, for two aerosol velocities, 1 m/s and 0.7 m/s, respectively. The filtration efficiency was determined as the average value from several measurements. The fractional filtration efficiency was determined in the particle size range from 225 nm to 3 µm. The overall filtration efficiency of PVDF nanofibrous filters in a particle size range above 300 nm was about 92.1 ± 2.3% and 89.5 ± 3.1%, for a gas velocity of 1 m/s and 0.7 m/s, respectively. The overall number filtration efficiency in the entire measured size range was 90.1 ± 2.2%, for a velocity of 1 m/s, and 87.1 ± 3.1% for a velocity of 0.7 m/s. The filtration efficiency was 91.0 ± 2.9%, for particles of 400 nm in diameter and for a gas velocity of 1 m/s, and it was 89.0 ± 4.1% for 0.7 m/s.

**Figure 15:**
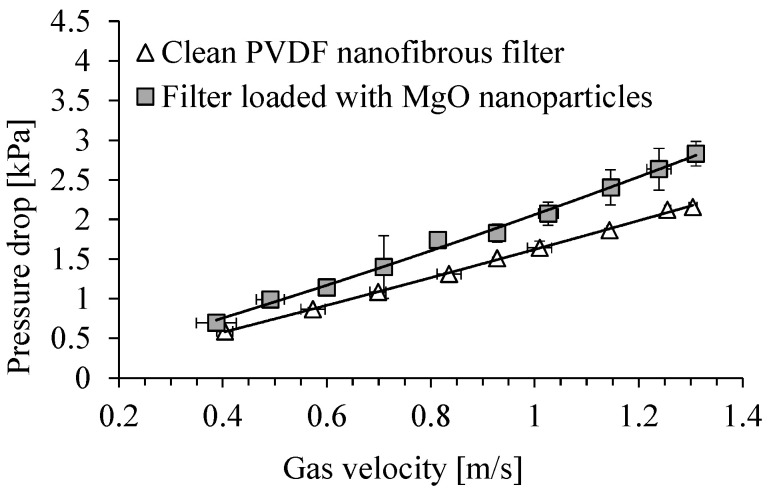
Dependence of pressure drop on PVDF nanofibrous filter on the gas velocity. Nanofibrous filter with Al_2_O_3_ particles after 5 min of filter operation. Total filter load 80 mg/m^2^.

The dependence of the pressure drop across the clean PVDF nanofibrous filter on the gas velocity is shown in Figure 15. The pressure drop for the same filter contaminated with Al_2_O_3_ particles, after 5 min of its operation, is shown in the same figure. The pressure drop was in the range of 1.8–1.9 kPa for a gas velocity of 1 m/s in both cases, clean PVDF nanofibrous filter and filter loaded with Al_2_O_3_ particles. For a velocity of 0.7 m/s the pressure drop was also similar for both of these cases, and was about 1.3 kPa. There was only a slight increase in the filter resistance as a result of filling the space between the filter fibers. This small difference in pressure drop for clean and loaded filter indicates that the inter-fiber spaces of the filter are poorly filled with the particles.

**Figure 16:**
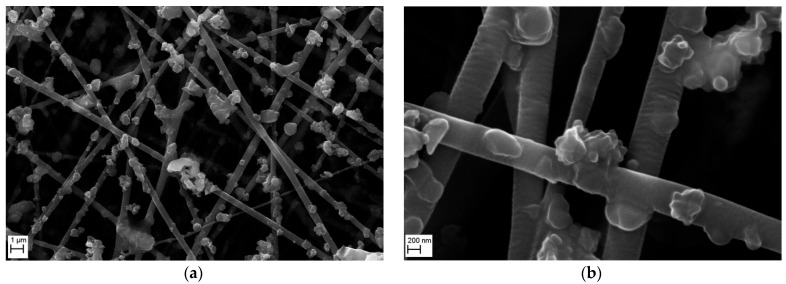
SEM micrographs of the surface of PVDF nanofibrous filter with Al_2_O_3_ particles after 5 min of filter operation for different magnifications: (**a**) 10,000×, (**b**) 50,000×. Total filter load 80 mg/m^2^. The fibrous filter is uniformly covered with the particles. Only single Al_2_O_3_ particles are deposited on the fibers with a small number of agglomerates. All voids remain empty.

Figure 16 shows the morphology of the nanofibrous filter covered with Al_2_O_3_ particles, examined by SEM after 5 min of operation, at two magnifications. The image obtained at a magnification of 10,000× (Figure 16a) shows that Al_2_O_3_ particles are mainly deposited on fibers of the filter. Only single particles are deposited on the fibers, and the number of aggregates or clusters is only a few. The micrograph obtained at 50,000× magnification (Figure 16b) confirms that the nanofibrous filter had slightly larger spaces between the fibers that resulted in a lower flow resistance of the filter. The Al_2_O_3_ particles did not fill the spaces between the fibers.

## 4. Discussion

The filtration efficiency of PVDF nanofibrous filters was measured using three types of metal oxide nanoparticles as the test particles: TiO_2_, MgO, and Al_2_O_3_. The PVDF nanofibrous filter was produced by the method of electrospinning, and the fibers were deposited onto a stainless steel scaffold. The production time of a single filter was 10 min. The mass of nanofibrous PVDF filter deposited in this time was estimated to be about 2.2 mg (13 g/m^2^). The diameter of PVDF nanofibers examined under SEM was in the range from 150 nm to 850 nm. The thickness of the investigated filters was intentionally limited to a couple of tens of nanofiber layers in order to determine the morphology of individual particles deposited onto the fibers in the process of filtration by a short time of loading. For these reasons, the obtained results of filtration efficiency cannot be compared with commercial filters (for example, HEPA filters) of a thickness of at least a couple of mm versus the investigated nonwoven filter of a thickness of 12 µm.

The measurements of filtration efficiency were carried out in a vertical channel for two aerosol flow rates, corresponding to a gas flow velocity of 1 m/s and 0.7 m/s. The average particles filtration efficiency up to 96.4% was obtained for a velocity of 1 m/s, in the case of TiO_2_ particles. The filtration efficiency of the PVDF nanofibrous filters decreased to a level lower than 80% for the particles in the size range below 300 nm. The filtration efficiency for smaller particles (<225 nm) could not be measured by the optical device used. The lowest overall and average mass filtration efficiency were obtained for MgO nanoparticles.

Table 1 summarizes the overall and average mass filtration efficiency of the PVDF nanofibrous filter for TiO_2_, MgO, and Al_2_O_3_ for a gas velocity of 0.7 m/s and 1 m/s in the measured particle size range. The average mass filtration efficiency for the investigated nanoparticles was about 88% and 90.0% for a gas velocity of 0.7 m/s and 1 m/s, respectively.

Before the measurement of filtration efficiency of each nanofibrous filter, the dependence of the pressure drop versus air velocity was determined for the clean filter. It can be noticed that in all cases, the pressure drop on the nanofibrous filter changes linearly with the air velocity that indicates the laminar flow through the nanofibrous filter. In order to determine an increase in the resistance of a filter, the pressure drop was measured after filter loading by a time of 5 min. As a result of filling the space between the fibers, a slight increase in the filter resistance occurred.

An increase in the pressure drop resulted from the deposition of particles on the fibers and in the voids between the fibers, but this process was dependent on the type of particle. The TiO_2_ and MgO particles formed aggregates of various shapes, which adhered to individual fibers or filled the voids between the fibers, or bridged the two neighbor fibers. These bridges caused an increase in the pressure drop across the filter after its contamination. In the case of Al_2_O_3_ particles, an increase in the pressure drop after filter contamination was smaller, only 0.2 kPa, by 1 m/s of the gas velocity, than for other particles. SEM images show that Al_2_O_3_ particles were deposited as single particles, with only a small number of clusters of various shapes adhering to individual fibers, and remaining free space between the fibers. The lack of clusters and bridges can result from small cohesion forces between Al_2_O_3_ particles, which are smaller than the aerodynamic force on these particles. The disintegration of the clusters causes a large number of particles to penetrate the filter.

Usually five mechanisms of particle deposition are considered for the understanding of the filtration process: the interception effect when the distance between the center of spherical particle and the axis of cylindrical fiber is equal to the sum of their radii, inertial impaction when the particle impacts the fiber due to its inertia, diffusion deposition when the particle moves randomly in collision with gaseous molecules that can lead to its impaction on the fiber, gravity effect when the particle falls under the action of gravitational force, and electrostatic interaction, such as Coulomb attraction, image or polarization forces, when at least one, the particle or fiber, is charged. The last mechanism is the most complex one. These mechanisms are usually referring to a single fiber and a single particle.

It can be assumed, in the investigated case, the main mechanisms responsible for the particle deposition are the interception and inertial impaction, because of relatively high velocity of the particles, and the diffusion due to the Brownian motion because of a small size of the particles, comparable with the mean free path of gaseous molecules. The inertial impaction can be confirmed by higher collection efficiency for higher air velocity. Gravitational deposition is usually excluded in the case of nanoparticles due a small magnitude of the gravitational force on them. Gravitational deposition can be effective for particles much larger than 1 µm [116]. Besides, in the investigated vertical configuration with the upwards flow, the gravity opposes the particle motion toward filter and cannot participate in the deposition. Electrostatic effects can also be plausible in the case of this filter, although neither the fibers nor the particles were intentionally electrically charged. However, the triboelectrification during the gas flow throughout the fibrous filter or the particle motion in the air cannot be excluded. These effects deserve separate studies.

It should be noted that the separation and accumulation of particles, apart from increased flow resistance through the nanofibrous filter, can also cause an increase in apparent separation surface of the fibers, which can lead to increased dust filtration efficiency.

## 5. Conclusions

The results of experimental studies on the filtration properties of PVDF nanofibrous filter used for the cleaning of air from various types of nanoparticles produced by different nanotechnology processes were presented in this article. The novelty of the presented research is the study of the effect of the type of particles on air filtration efficiency and the morphology of nanoparticles deposited on nanofibers. Highly porous nanofibrous filters produced by electrospinning of polymer consist of the fibers with a diameter of 150 to 850 nm. Filters produced by the method of electrospinning can be uniform and composed of nanofibers of similar diameters, which cause the same pressure distribution over the entire filter surface and uniform filling of filtered particles. The results of experimental studies showed that nanofibrous filter made of a thin nanofibrous material of surface density of 13 g/m^2^ has a high filtration efficiency for nano- and submicron particles. The filtration efficiency of a nanofibrous filter of a thickness of 12 µm can be higher than 90%, for particles larger than 300 nm, but decreases to about 80% for particles in the range of 300 down to 225 nm, for a gas velocity of 1 m/s. The pressure drop was smaller than 2.0 kPa for this velocity. Particles smaller than 225 nm have not been measured by the used particle size spectrometer.

## Figures and Tables

**Table 1:** Summary comparing filtration efficiencies of TiO_2_, MgO, and Al_2_O_3_ for gas velocity of 0.7 m/s and 1 m/s.

Nanoparticles	TiO_2_		MgO		Al_2_O_3_	
Gas flow velocity [m/s]	0.7	1 m	0.7 s	1	0.7 s	1
The overall filtration efficiency [%]	84.8 ± 6.7	89.6 ± 4.4	82.9 ± 1.5	83.8 ± 2.7	87.1 ± 3.1	90.1 ± 2.2
Average mass filtration efficiency [%]	88.7 ± 6	92.5 ± 3.9	88.6 ± 0.8	89.9 ± 2.2	88.8 ± 3.1	91.5 ± 2.3

## Data Availability

Data available upon request.

## Abbreviations

The following abbreviations are used in this manuscript:

DMAC	Dimethylacetamide
PMMA	Poly(methyl methacrylate)
PVDF	Polyvinylidene fluoride
